# Illicit Drug Use Among Gym-Goers: a Cross-sectional Study of Gym-Goers in Sweden

**DOI:** 10.1186/s40798-017-0098-8

**Published:** 2017-08-29

**Authors:** Yasmina Molero, Ann-Sofie Bakshi, Johanna Gripenberg

**Affiliations:** 1 STAD, Centre for Psychiatry Research, Department of Clinical Neuroscience, Karolinska Institutet, & Stockholm Health Care Services, Stockholm County Council, Norra Stationsgatan 69, SE-1113-64 Stockholm, Sweden; 2 Department of Medical Epidemiology and Biostatistics, Karolinska Institutet, Stockholm, Sweden

**Keywords:** Illicit drug use, Gym-goers, Anabolic-androgenic steroids, Cross-sectional study, Drug prevalence

## Abstract

**Background:**

The use of anabolic-androgenic steroids has increased among gym-goers, and it has been proposed that this may be part of a polysubstance use pattern that includes the use of illicit drugs. Still, epidemiological data on illicit drug use among gym-goers of both genders are meager. The aim of the present study was thus to examine the use of illicit drugs and its correlates in a large sample of men and women who engaged in weight training at gyms across Sweden.

**Methods:**

In this cross-sectional study, a total of 1969 gym-goers who engaged in weight training in 54 gyms across Sweden were invited to fill in a questionnaire. The questionnaire included 25 items on background variables, weight training frequency, use of illicit drugs and doping substances, and non-medical use of benzodiazepines.

**Results:**

Of the gym-goers, 19.6% reported having ever used illicit drugs, 6.5% reported use during the past 12 months, and 2.1% during the past 30 days. The most commonly used drug was cannabis, followed by cocaine, amphetamine, and ecstasy. Almost 40% of those who reported drug use had used more than one drug. Male participants and participants between 20 and 39 years of age made up the majority of users. Furthermore, 5.1% of the reported drug users had ever used a doping substance. There was an almost threefold higher odds (OR = 2.99, 95% CI = 1.16–7.66, *p* < 0.023) of doping use among people who had reported drug use as compared to non-users.

**Conclusions:**

Training at gyms is typically considered a health-promoting behavior. However, our results revealed a slightly higher prevalence of illicit drug use among gym attendees as compared to the general population. Our findings may have captured an underrecognized group of young adult males who engage in weightlifting and use illicit drugs recreationally and/or as training aids. Developing knowledge is imperative in orientating preventive efforts among at-risk gym-goers.

**Trial Registration:**

ISRCTN11655041

## Key Points

- Approximately one in five gym-goers report use of illicit drugs, most commonly cannabis and stimulants. The rates of drug use are higher among young adult males.
- Illicit drug use appears to be associated with the use of doping substances.
- Gyms could provide an innovative setting for intervention and prevention efforts targeting doping and illicit drug use, because such establishments already deal with health promotion.

## Background

Research shows that 65% of citizens in the European Union exercise at least once a week. Among this group, 30% exercise at sport clubs such as gyms and fitness centers [1]. In the USA, approximately 21% of adults reported exercising regularly [2], and more than 55 million memberships were purchased at health clubs and fitness centers in 2015 [3]. Exercise is a health-promoting activity associated with several benefits, including reduced risk of coronary heart disease, type 2 diabetes, breast and colon cancer, as well as premature mortality [4]. At the same time, there is growing evidence indicating that use of anabolic-androgenic steroids and non-medical use of prescription drugs have increased among gym-goers [5–7]. It has been proposed that this may be part of a polysubstance use pattern that involves other illicit drugs as well (e.g., cannabis and stimulants) [8]. Polysubstance use is associated with poorer mental health, sexual risk behavior, negative social consequences, and increased risk of infectious disease [9, 10]. Furthermore, concurrent use of substances may have synergistic negative effects on brain function [9]. It has been suggested that doping prevention efforts should target gym-goers [11]. Given the association between doping substances and illicit drugs [8, 12–14], prevention efforts could therefore also address the use of illicit drugs [6]. Still, the research base on illicit drug use among gym-goers is limited, and developing knowledge is imperative in orientating preventive efforts.

Several studies have examined the use of illicit drugs among sportspeople through questionnaires or toxicological testing [15–23]. Results from these studies indicate elevated rates of illicit drug use, with cannabis and stimulants being the most commonly used drugs. However, these samples have been restricted to elite athletes or adolescent populations. Epidemiological studies focusing on illicit drug use among adult gym-goers are few in number. One study on 311 gym-going gay men in New York showed that 6–35% (depending on the type of drug) reported having used a drug during the past 6 months [24]. Another study on 1592 gay men who attended gyms in London showed that 56% reported having used an illicit drug during the past year [25]. It was proposed that many of these men do not frequent gyms as a health-promoting activity, but rather to achieve an idealized muscular, physically strong body. This was suggested to be indicative of a gay subculture that focuses on physical prowess and risk behaviors, including illicit drug use [24]. However, results from these two studies may be difficult to generalize to other gym-attending populations and across gender.

The present study is part of a larger on-going project entitled 100% Pure Hard Training (100% PHT) [26]. In the 100% PHT project, the prevalence of doping substances (i.e., anabolic-androgenic steroids and growth hormones) and other illicit drugs is measured among gym-goers who engage in weight training (i.e., work with free weights or machines), and the effect of a doping prevention programme (i.e., 100% PHT) is examined. In the present study, the use of illicit drugs, benzodiazepines, and doping substances among gym-goers was assessed using a cross-sectional design. This assessment was carried out prior to implementation of the prevention programme.

The overall aim of the study is to examine the use of illicit drugs in a large sample of men and women who engage in weight training at gyms across Sweden. Specifically, we conducted a cross-sectional examination of the (a) frequency and type of illicit drugs used, (b) frequency of non-medical use of benzodiazepines, (c) age and sex differences in illicit drug use, (d) associations between use of illicit drugs and weight training frequency, and (e) associations between use of illicit drugs and use of doping substances.

## Methods

### Study Design

The present study has a cross-sectional study design.

### Participants and Procedure

In the spring of 2015, a questionnaire was distributed to gym-goers at 54 gyms in Sweden. The gyms were recruited either through Prevention of Doping in Sweden (PRODIS; a national network comprising governmental agencies, universities, county administrative boards, municipal prevention coordinators, representatives from the police force and gyms, the Swedish Sports Confederation, and the Swedish Anti-Doping Hotline) or through the research centre STAD (Stockholm Prevents Alcohol and Drug Problems). The questionnaire was distributed prior to implementation of the doping prevention programme 100% PHT at the gyms. During data collection, research staff stood by the entrance of the gyms on a weekday afternoon or evening and asked arriving gym-goers above 16 years of age whether they were going to do weight training (i.e., work with free weights or machines). Those who replied in the affirmative were invited to participate in the study. Gym-goers who agreed to participate were asked to complete a questionnaire. To guarantee anonymity, participants completed the questionnaire anonymously and then placed it in an envelope and sealed it, before returning it to the research staff. For the study, 2631 gym-goers were asked to participate, and 1969 of them agreed (74.8% response rate). All gyms were located in urban areas; 39% of the gyms were located in a major city (i.e., more than 200,000 inhabitants), 37% in a large town (i.e., more than 50,000 inhabitants), and 13% in a small town (i.e., more than 15,000 inhabitants) [27]. For more details on the data collection, please see study protocol [26].

### Measures

The questionnaire distributed to the participants was based on another questionnaire previously used in a Swedish study measuring anabolic-androgenic steroid use among gym-goers [28]. It included 25 items on background variables, weight training frequency, use of illicit drugs, doping substances, and nutritional supplements, as well as attitude items on doping prevention. The questionnaire took 5 to 10 min to complete. The following measures were included in the current study:

#### Demographic Factors

Participants were asked to report their age, sex, employment, and highest educational attainment.

#### Weight Training Frequency

Participants were asked “*How often do you do weight training at the gym*?” Response alternatives were never, less than once a week, once or twice a week, three or four times a week, and five or more times a week.

#### Use of Illicit Drugs

Participants were asked “*Have you ever used any illicit drugs or prescription drugs without a doctor’s order?*” Participants were then asked to specify the type of illicit drugs that they had used. Response alternatives were cannabis, amphetamines, cocaine, heroin, hallucinogens, ecstasy, benzodiazepines, or other. Furthermore, participants were asked “*Have you used any illicit drugs or prescription drugs without a doctor’s order during the past 12 months?*” and “*Have you used any illicit drugs or prescription drugs without a doctor’s order during the past 30 days?*”

#### Use of Doping Substances

To assess the use of doping substances, participants were asked “*Have you ever used any anabolic-androgenic substances, growth hormones or other doping substances without a doctor’s order*?”

### Statistical Analysis

First, frequencies and proportions of demographic factors and weight training intensity are presented for the whole sample (*n* = 1969). Illicit drug use and associated characteristics are also presented for the whole sample. In a subsequent step, drug characteristics are presented only for those who reported illicit drug use (*n* = 385). Logistic regression models were then calculated in the whole sample (*n* = 1969) to examine associations between illicit drug use and weight training frequency, as well as associations between illicit drug use and use of doping substances. Crude odds ratios (OR) and 95% confidence intervals (95% CI) are presented. Odds ratios were then adjusted; when examining associations between illicit drug use and weight training frequency, odds ratios were adjusted for age and sex. When examining associations between illicit drug use and the use of doping substances, odds ratios were adjusted for age, sex, weight training frequency, and number of illicit drugs used. IBM SPSS Statistics 24.0 was used in all analyses. The STrengthening the Reporting of OBservational studies in Epidemiology (STROBE) guidelines were followed (S1).

### Power Analysis

Previous studies have shown that the effect of prevention programmes is rather low, typically about 15–20% [29, 30]. To measure the intervention effect, a power analysis was carried out a priori; a minimum of 40 gyms (20 intervention gyms and 20 control gyms) and a minimum of 1600 participants (800 individuals per condition) were required at each data collection point to achieve a power of 80% at an alpha level of .05 (two-tailed). In the current pre-intervention study, 27 intervention gyms and 27 control gyms have been enrolled, and a total of 1969 individuals completed the questionnaire.

### Ethical approval

The present study adheres to the criteria of the World Medical Association Declaration of Helsinki, and has been approved by the regional ethical review board of Karolinska Institutet, Sweden (registration number: 2016/142-31/3).

## Results

The present study included 1969 gym-goers from 54 gyms. The sample consisted of a large proportion of males (65.3%), and the majority of participants were below 40 years of age. Most gym-goers were employed, had completed secondary or tertiary education, and reported regular weight training. In the total sample of gym-goers, 2.0% (40) reported having ever used a doping substance (Table 1).

**Table 1:** Sample characteristics of gym-goers (*n* = 1969)

Sex^a^	
Male	63.5% (1251)
Female	35.8% (705)
Age^b^	
16–19 years	9.3% (184)
20–29 years	41.2% (811)
30–39 years	19.0% (375)
40–49 years	14.3% (281)
50–59 years	9.9% (194)
60 years and older	5.8% (114)
Occupation^c^	
Employed	71.4% (1406)
Unemployed	3.1% (62)
Studying	19.2% (378)
Retired	2.5% (49)
Sick leave	0.8% (16)
Other	2.1% (42)
Highest educational attainment^c^	
Primary education	8.2% (162)
Secondary education	42.7% (841)
Tertiary education	31.4% (619)
Weight training frequency^c^	
Never	1.1% (22)
Less than once a week	3.8% (75)
Once or twice a week	23.9% (470)
Three to four times a week	47.6% (937)
Five or more times a week	23.5% (463)
Lifetime use of doping substances	
Has ever used a doping substance	2.0% (40)

^a^Missing information for 13 individuals

^b^Missing information for 10 individuals

^c^Missing information for 16 individuals

Among the participating gym-goers, 19.6% (385) reported having ever used an illicit drug or benzodiazepines without a prescription (Table 2). Furthermore, 6.5% reported that they had done so during the past 12 months and 2.1% during the past 30 days. Cannabis was the most commonly used illicit drug and had been used by 92.2% of participants reporting illicit drug use. Between 20 and 25.2% of the reported illicit drug users had used cocaine, amphetamine, and/or ecstasy. Heroin was the least common illicit drug. Among those who reported illicit drug use, 17.4% reported having used two drugs and 21.3% reported having used three or more drugs (Table 3). The prevalence of individuals who reported a lifetime use of doping substances was higher among those with prior illicit drug use (5.1%) than in the total sample. There were statistically significant gender differences in prevalence rates; males demonstrated higher rates of cannabis, amphetamine, cocaine, and ecstasy use. Males also demonstrated significantly higher rates of polysubstance use. Furthermore, there were statistically significant age differences; younger individuals (below age 40) demonstrated higher prevalence rates of cannabis, amphetamine, cocaine, hallucinogen, and ecstasy use and also higher rates of polysubstance use (Table 3).

**Table 2:** Lifetime drug use among gym-goers (*n* = 1969)

Lifetime drug use	
Has ever used drugs	19.6% (385)
Sex^a^	
Male	71.5% (274)
Female	28.2% (108)
Age^b^	
16–19 years	7.8% (30)
20–29 years	49.7% (191)
30–39 years	26.6% (102)
40–49 years	6.5% (25)
50–59 years	5.5% (21)
60 years and older	3.9% (15)
Recent illicit drug use	
Has used drugs during the past 12 months	6.5% (128)
Has used drugs during the past 30 days	2.1% (42)

^a^Missing information for three individuals

^b^Missing information for one individual

**Table 3:** Drug characteristics among gym-goers who reported lifetime drug use (*n* = 385)

	All drug users	Males^a^	Females^a^	*χ* ^2^	*p*	Age 16–39^a^	Age 40 and over^a^	*χ* ^2^	*p*
Lifetime use of illicit drugs									
Cannabis	92.2% (355)	67.0% (258)	24.7% (95)	16.04	< 0.001	77.4% (298)	15.8% (56)	41.71	< 0.001
Cocaine	25.2% (97)	19.5% (75)	5.7% (22)	8.07	0.018	21.8% (84)	3.4% (13)	13.48	< 0.001
Amphetamine	21.8% (84)	16.1% (62)	5.5% (21)	10.47	0.005	17.7% (68)	4.2% (16)	5.07	0.024
Ecstasy	20.0% (77)	14.3% (55)	5.5% (21)	9.28	0.010	18.4% (71)	1.6% (6)	18.91	< 0.001
Hallucinogens	13.0% (50)	9.6% (37)	3.4% (13)	2.33	0.312	11.4% (44)	1.3% (5)	9.43	< 0.001
Benzodiazepines	8.6% (33)	6.8% (26)	1.8% (7)	3.26	0.196	7.0% (27)	1.6% (6)	2.25	0.133
Heroin	1.6% (6)	1.6% (6)	0.0% (0)	3.41	0.182	0.8% (3)	0.8% (3)	1.14	0.286
Other	3.1% (12)	2.9% (11)	0.3% (1)	4.05	0.132	2.6% (11)	0.3% (1)	2.71	0.100
Number of illicit drugs used (lifetime)									
One drug	59.7% (230)	41.3% (159)	18.2% (70)	15.55	0.001	48.3% (186)	11.4% (44)	41.73	< 0.001
Two drugs	17.4% (67)	13.5% (52)	3.9% (15)			14.8% (57)	2.3% (9)		
Three or more drugs	21.3% (82)	15.8% (61)	5.2% (20)			19.0% (73)	2.6% (10)		
Lifetime use of doping substances									
Doping substances	4.9% (19)	6.4% (17)	1.9% (2)	3.05	0.218	5.4% (17)	3.4% (2)	0.40	0.529

^a^Missing information for one individual

A logistic regression was performed to examine the relationship between weight training frequency and illicit drug use (Table 4). No statistically significant associations were found, neither in the crude analyses, nor when adjusting for sex and age. Another logistic regression was then performed to assess the relationship between illicit drug use and use of doping substances. The logistic regression model was statistically significant (*χ* ^2^ = 18.391, *p* < .001, *df* = 1), showing that individuals who reported illicit drug use were more than four times more likely to use doping substances compared to individuals without prior illicit drug use. When adjusting for age, sex, weight training frequency, and number of illicit drugs used, the model remained statistically significant (*χ* ^2^ = 38.514, *p* < .001, *df* = 5). Results showed that individuals who reported illicit drug use were almost three times more likely to use doping substances compared to individuals without prior illicit drug use.

**Table 4:** Associations between illicit drug use and weight training frequency and use of doping substances, respectively (*n* = 1969)

	Crude			Adjusted		
	OR	95% CI	*p*	OR	95% CI	*p*
Weight training frequency	1.07	0.94–1.23	0.296	0.90^a^	0.78–1.04	0.143
Use of doping substances	4.40	2.28–8.46,	*p* < 0.001	2.99^b^	1.16–7.66	*p* < 0.023

*OR* odds ratio 95%, *CI* 95% confidence intervals

^a^Adjusted for sex and age

^b^Adjusted for sex, age, weight training frequency, and number of drugs used

## Discussion

### Discussion

In the present study, 1969 gym-goers at 54 gyms in Sweden completed a questionnaire covering the use of illicit drugs and prescription drugs. Results showed that 19.6% of gym-goers reported having ever used illicit drugs. Moreover, of this group, 6.5% reported use during the past 12 months and 2.1% during the past 30 days. Among those who reported illicit drug use, the most commonly used illicit drug was cannabis, followed by cocaine, amphetamine, and ecstasy. Furthermore, 5.1% of those reported a lifetime use of doping substances. Almost 40% of those reporting illicit drug use reported polysubstance use. Furthermore, male participants and participants between 16 and 39 years of age demonstrated significantly higher rates of illicit drug use and polysubstance use than female participants and participants above age 40, respectively.

Our findings are in line with two Swedish population-based surveys. In a survey from 2008/2009, 18% of respondents reported having used illicit drugs on at least one occasion in their lives [31]. In a survey from 2012, 3.1% of respondents had used an illicit drug during the past 12 months and 1.1% during the past 30 days [32]. Training at gyms is typically a health-promoting behavior; hence, it could be hypothesized that the estimates in our sample would be lower than the rates found in the general population. However, our results pointed to a slightly higher prevalence of illicit drug use among gym-goers. The elevated rates in our study could be explained by age and sex differences in the samples; our study included more males and more individuals from younger age categories, where illicit drug use is generally more prevalent [31, 32].

Conversely, participants in the present study reported a lower lifetime prevalence of illicit drug use as compared to the European average. In the European Union, approximately 25% of 15- to 64-year-olds are estimated to have used an illicit drug [33]. Studies consistently indicate lower estimates of illicit drug use in Sweden compared to many countries [34–36], which also could be reflected among gym-goers [24, 25]. Further research is needed to specifically examine the use of illicit drugs and prescription drugs (beyond benzodiazepines) in gym settings and to assess between-country differences as well as associations with demographic factors. Prevalence rates in our study were higher than rates among elite athletes in Europe and Australia [17, 18, 21]. Higher estimates of illicit drug use among gym-goers might be expected, however, as elite athletes generally have a healthier lifestyle and are also subject to anti-doping tests, which may have a deterrent effect on illicit drug use [18].

Two studies have examined the use of illicit drugs among gay male gym-goers in New York and London, respectively. In these studies, prevalence rates were markedly higher than in our study; in the New York study, estimates ranged between 6% (for hallucinogen use) and 35% (for inhalant nitrite use) during the past 6 months [24]. Among the men recruited from London gyms, 56% reported having used an illicit drug during the past 12 months [25]. It was proposed that these studies largely reflected a specific subculture among gay and bisexual men who frequented clubs and engaged in risk behaviors (i.e., substance use and sexual risk behavior), which could explain the higher prevalence rates.

The most commonly used illicit drugs in our study were cannabis, cocaine, amphetamine, ecstasy, and hallucinogens—drugs that can be used both as training aids or “body image drugs,” or recreationally as so-called club drugs [37–40]. Moreover, approximately 40% of the illicit drug users in our sample reported polysubstance use, and the rates were significantly higher among younger participants and males. Our results could also have captured a group of young adult males who focus on physical appearance and engage in partying and risk behaviors, comparable to the gay men in the gym studies discussed above. It has been suggested that drug use practices and weight training in a male “body subculture” can be understood as a way to construct a masculine identity and to achieve an idealized male body [41]. Our estimates were higher than the estimates in a study of anabolic substance use among 611 visitors to German fitness centers, where 15.9% reported using other illicit drugs [42]. Almost all participants in our study engaged in weight training, as opposed to 25% of the respondents in the German study. Again, the higher prevalence rates in our study could support the notion of a physical achievement-oriented group who engage in weight training and use illicit substances for ergogenic and/or recreational purposes.

In our study, however, we found no associations between weight training frequency and illicit drug use. Prior research on the association between physical activity and illicit drug use is mixed: Some studies suggest that the degree of sporting activity is negatively associated with substance use [43]. Others point to a curvilinear link between sporting activities and illicit drug use, i.e., that inactive and intensive levels are associated with greater use of illicit drugs than moderate levels are, particularly for strength sports (weightlifting and body-building) [19]. However, these studies have largely been based on adolescent populations, and it has been proposed that the relationship between physical activity and illicit drug use is affected by age [19], which could explain our differing results.

The overall prevalence of the lifetime use of doping substances (i.e., anabolic-androgenic substances and growth hormones) was lower in our total sample than in other gym samples [28, 42, 44–46]. Nonetheless, our results showed a threefold higher in odds of doping use among people who had reported illicit drug use as compared to non-users. Previous research has pointed to a strong relationship between use of doping substances and use of other illicit drugs [14, 47–49]. Illicit drugs may be used to increase or decrease the effects of anabolic-androgenic steroids (e.g., as pain relief to increase energy levels or to promote sleep) [6], and it has been proposed that doping prevention efforts should therefore address other illicit drugs as well [6]. The *European Commission Group of Experts on Anabolic Androgenic Steroid Use in Recreational Sports* has identified gyms as important target arenas for preventive efforts and has also suggested three key elements that should be included in research to inform policy, practice, and interventions: information on doping prevalence, use of other illicit drugs, and determinants and correlates [11].

### Strengths and Limitations

The present study has both strengths and limitations that should be taken into account when interpreting the findings. One important strength is that we included a large sample of men and women and that participants were recruited from 54 gyms across the country. Limitations include a possible underestimation of illicit drug prevalence rates due to a higher degree of attrition among individuals who use illicit drugs or to recollection bias, social desirability, or fear that reported illicit drug use may attract unwanted attention to the gym [50]. Males and individuals under age 40 made up a large proportion of participants, which could limit the generalizability of the findings. Furthermore, the questionnaire was distributed on weekday afternoons and evenings, and only individuals who engaged in weight training were invited to participate, which could limit generalizability further. Although the questionnaire has been used in a prior study [28], its psychometric properties have not been validated. Another limitation was that the study was cross-sectional; thus, we could not establish causality in the association between illicit drug and doping use.

## Conclusions

Our study examined illicit drug use in a large sample of male and female adult gym-goers. Illicit drug use estimates in our study were slightly elevated in comparison to estimates in population-based studies in Sweden [31, 32] and could simply reflect illicit drug use in the general population. This may seem contradictory, however, as training at gyms is typically considered a health-promoting behavior and prevalence rates could thus be expected to be lower among gym-goers. Our findings show that a substantial proportion of young adult males who lift weights have used several illicit drugs. This suggests that illicit drug use among sportspeople, possibly for ergogenic or analgesic purposes, is a public health problem not limited to elite athletes [17, 18, 21]. A proportion of younger recreational sportspeople may be at risk of developing substance abuse problems (including doping substances), yet there are few arenas on which young individuals can be reached other than nightlife settings and universities [50–52]. Previous research shows that young people who party frequent gyms to socialize, to offset the effects of substance use, or to purchase illicit drugs [53]. Gyms could thus provide an additional innovative setting for intervention and prevention efforts targeting doping and illicit drug use, because such establishments already deal with health promotion and do not allow on-site alcohol consumption.

## Abbreviations

100% PHT: 100% Pure Hard Training
95% CI: 95% confidence intervals
OR: Odds ratios
PRODIS: Prevention of Doping in Sweden
STAD: Stockholm Prevents Alcohol and Drug Problems
STROBE: STrengthening the Reporting of OBservational studies in Epidemiology guidance
